# Exercise adaptations and TGF-β1 levels in recreational cyclists

**DOI:** 10.1016/j.amsu.2021.102872

**Published:** 2021-09-22

**Authors:** Ida Ayu Eka Widiastuti, Aryadi Arsyad, Irfan Idris, Ilhamjaya Patellongi, Hamsu Kadriyan, Gede Wira Buanayuda, Dian Puspita Sari, Rohadi Muhammad Rosyidi

**Affiliations:** a Department of Physiology, Medical Faculty of Mataram University, Mataram, Indonesia; b Postgraduate Program, Medical Faculty of Hasanuddin University, Makassar, Indonesia; c Department of Physiology, Medical Faculty of Hasanuddin University, Makassar, Indonesia; d Department of Otolaryngology Head and Neck Surgery, Medical Faculty of Mataram University, Mataram, Indonesia; e Department of Biomedical Sciences, Medical Faculty of Mataram University, Mataram, Indonesia; f Department of Medical Education, Medical Faculty of Mataram University, Mataram, Indonesia; g Department of Neurosurgery Medical Faculty of Mataram University, West Nusa Tenggara General Hospital, Mataram, Indonesia

**Keywords:** Regular physical exercise, Adaptation, Transforming growth factor beta 1, Cyclists

## Abstract

**Background:**

Cycling is a physical exercise that is widely performed to improve physical fitness. Regular physical exercise will lead to adaptations to exercise. This adaptation is useful in suppressing the production of reactive oxygen stress (ROS) generated in response to cellular metabolism that uses oxygen. Transforming growth factor beta-1 (TGF-β1) plays a role in increasing the production of ROS, thus, when the concentration is low, it would lead to an improvement in physical fitness. This study aims to compare levels of TGF-β1 between recreational cyclists and sedentary groups. In addition, this research also compares several other parameters, which are fasting blood sugar levels and lipid profiles (triglycerides, total cholesterol, HDL cholesterol, and LDL cholesterol) between cyclists and sedentaries.

**Methods:**

This was an observational analytical study with a cross-sectional design. The research subjects consisted of 2 groups, each consisting of 21 participants, namely the recreational cyclist and the sedentary group. Anthropometric examinations were carried out, including body weight, height, body mass index, waist circumference, and body fat percentage. Fasting blood glucose concentration and lipid profile (Triglyceride – *TG*, Total Cholesterol – *Total C*, HDL Cholesterol – *HDL-C*, and LDL Cholesterol – *LDL-C*) were determined by the enzymatic colorimetric methods, and TGF-β1 levels were determined using the fluorescence of specific antibodies for TGF-β1 (pg/ml) using ELISA method. Statistical analysis was performed using IBM SPSS v. 25.

**Results:**

The anthropometric variables, other than body height, did not differ significantly between the two groups, so did the fasting blood glucose concentration. Nevertheless, the lipid profile (TG, Total C, HDL-C and LDL-C) were found to be significantly better in the cyclist group (p < 0.05).

The mean level of TGF-β1 in recreational cyclists was 8, 908.48 pg/ml, lower than the control group, 10, 229.28 pg/ml. The results of the unpaired *t*-test showed significant mean differences between the two groups, (p = 0.001; p < 0.05).

**Conclusion:**

The levels of TGF-β1 in the recreational cyclist group were lower than the sedentary group. Regular physical exercise will trigger exercise adaptations that can suppress latent TGF-β1 activation.

## Highlights

• This study aims to compare levels of TGF-β1 between recreational cyclists and sedentary groups
• The research subjects consisted of 2 groups, each consisting of 21 participants, namely the recreational cyclist and the sedentary group.
• Anthropometric examinations were carried out, including body weight, height, body mass index, waist circumference, and body fat percentage
• The levels of TGF-β1 in the recreational cyclist group were lower than the sedentary group

## 1 Introduction

Regular, moderate-intensity physical activity, such as walking, or cycling to work, and participating in sports has significant health benefits. Evidence has concluded that regular physical activity contributes to primary and secondary prevention of several chronic diseases and is also associated with a reduced risk of premature death [1].

Cycling is a popular physical activity that is also considered as a recreational sport, which purpose is to improve physical fitness, bring fun, and promote social involvement. Cycling has many benefits to health. In children and adolescents, cycling can improve cardiorespiratory and muscle fitness, help to obtain ideal body composition, and improve bone health. In adults, it has been showed that cycling can reduce premature death, heart disease, type II Diabetes Mellitus, abnormal lipid profile, high blood pressure, metabolic syndrome, as well as colon and breast cancer. Furthermore, in adults, cycling can anticipate weight gain due to increased use of energy reserves in the form of glycogen and triglycerides, support weight loss when combined with diet, improves cardiovascular and muscle fitness, decrease depression, and in older adults, cycling can help to maintain better cognitive function [2]. A cohort study conducted in Copenhagen Denmark involving 13.375 female and 17.265 male respondents and followed for 14.5 years concluded that cycling to work reduces all causes of death by 28% [3].

Physical exercise is a natural source of reactive oxygen species (ROS) [4]. ROS production is believed to be the underlying mechanism of an array of biochemical reaction and physiological response during an exercise and is an indication of the presence of oxidative stress. There is a strong evidence that ROS has a role in cell and tissue injury induced by physical exercise but at the same time also stimulate antioxidant defense through the role of antioxidant enzymes [5].

Various studies have shown that transforming growth factor-beta 1 (TGF-β1) can increase the production of mitochondrial ROS in various cell types [6]. TGF-β1 is one of the 3 TGF-β isoforms, which plays a role in cell proliferation, immune function, and the production of Extracellular Cell Matrix (ECM). Circulating levels of TGF-β1 provide potential use as a prognostic marker of certain diseases [7].

Currently, many recreational sports communities are developing in the society, especially in urban areas, and one of which is the cycling communities. These groups typically perform regular cycling activities for 2–3 times a week, on average 30–60 min each time. However, regular physical exercises will induce an adaptation which then is able to increase the function of all organs as well as mitochondrial biogenesis and antioxidant defences, and at the same time, reduce oxidative damage [8].Thereis a gap in the literature on the relationship of TGF-β1 level with adaptation induced by physical activity in recreational cyclists.

## 2 Methods

### 2.1 Setting

This study was carried out in Mataram City, West Nusa Tenggara Province, Indonesia. Mataram City is located in Lombok Island, 2 h flight from the capital city Jakarta. According to the Central Bureau of Statistics of Mataram City, 495.681 people lived in this city of 61.30 km square. There were more than 20 cycling communities in Mataram City, each community is usually characterized by specific types of bicycle used, i.e. road bike, mountain bike, or roadster bicycle.

### 2.2 Participants

This was an observational analytical study with a cross-sectional design. Forty-two participants were involved in this study and divided into two groups: the first group consisted of 21 male, recreational cyclists in Mataram City, West Nusa Tenggara Province who have been actively cycling for at least one year. The participants in this group were recruited from three bicycle communities in Mataram City. The criteria for recreational cyclists in this study were (1) performing regular cycling activities ≥1 time/week (2) cycling ≥1 h/week, and (3) cycling ≥20 km/week. The second group consisted of 21 male subjects of similar age range with the first group (18–65 years old) and rarely involved in physical exercise (sedentary lifestyle). Subjects who have a history of cardiopulmonary disease, malignancy, or currently on treatment using immunosuppressant agents were excluded because these can affect the level of TGF-β1. All the participants were given information about the study before being asked to give their consent to participate. This study has received approval from the Ethical Committee of the Faculty of Medicine, UniversitasMataram (248/UN18.F7/ETIK/2019).

### 2.3 Variables

The predictor variable in this study was the adaptation to physical activity, particularly being a recreational cyclist with the selection criteria mentioned above. The criterion variable was the level of TGF-β1. Participants' identity was collected using structured interviews. In addition, this study also obtained participants' demographic and anthropometric data, lipid profile and fasting blood glucose (FBG) concentration. Participants' age was recorded in years. Anthropometric data were obtained by taking measurements which included: body height (in centimeter) using Microtoise, waist circumference (in centimeter) with a meter, body weight (in kilogram), body mass index (kg/m2), body fat percentage, and visceral fat were measured digitally using the Omron® HBF-375's body composition monitor. Assessment of lipid profile in this study include Triglyceride – *TG*, Total Cholesterol – *Total C*, HDL Cholesterol – *HDL-C*, and LDL Cholesterol – *LDL-C*. Participants were required to fast for 10–12 h before blood sample for the lipid profile and FBG concentration assessments were taken. The enzymatic colorimetric methods were used to determine the lipid profile and FBG concentration.

### 2.4 Blood collection and measurement of TGF- β1

Blood samples were collected in the laboratory of Mataram University Hospital by the trained laboratory workers. The night before the day of blood collection, subjects were required to fast for 10–12 h, without doing strenuous activities. Subject was still allowed to drink water ad libitum. Blood sample was taken in the morning between 08.00 and 09.00 a.m. A 3-cc blood sample was obtained from the antecubital vein. Subsequently, blood samples were centrifuged in order to obtain the serum. Measurement of serum transforming growth factor-beta 1 (TGF-β1) levels was carried out using the ELISA method with serum TGF-β1 (AbClonal®) kit. The ELISA examination procedure was carried out according to the protocol in the kit. Serum TGF-β1 levels were determined from the fluorescence of specific antibodies for TGF-β1 (pg/ml).

### 2.5 Statistical analysis

Mean values and standard deviations were presented for all measurement variables. Unpaired T-Test was used to compare means between the cyclist and the sedentary group if the data were normally distributed, otherwise the Mann-Whitney test was used. The Shapiro-Wilk test was performed to determine the normality of the data distribution and the Levene test for data homogeneity. Statistical analysis to compare the level of TGF-β1 between the recreational cyclist group and sedentary group was carried out using Unpaired T-test.

## 3 Results

As presented in Table 1, the anthropometric characteristics of the subjects did not differ between the cyclist group and the sedentary group, except for body height that was found to be higher among the cyclists (p = 0.039). The two groups also did not differ significantly in terms of FBG concentration. However, the lipid profile of the cyclist group was significantly better compared to the sedentary group. The first group showed lower TG, Total C, and LDL-C, and higher HDL-C (all p < 0.01).

**Table 1:** Subject characteristics.

Subject characteristics	Mean ± SD		Min-max value		p
	Cyclists	Sedenter	Cyclists	Sedenter	
Age	40 ± 11.15	39.3 ± 8.53	20–64	27–57	0,817^a^
Weight (kg)	65.13 ± 8.92	61.68 ± 11.12	52.3–82.6	43–89	0,275^a^
Height (cm)	166.78 ± 5.24	162.98 ± 6.26	158.6–17.5	147–175	0,039^a^
Body mass index (kg/m2)	23.45 ± 2.68	23.16 ± 3.60	18.8–27.8	16.4–31.7	0.765^a^
Body fat percentage (%)	21.20 ± 5.09	21.73 ± 6.05	11.8–29.8	9.9–33.2	0.759^a^
Waist circumference (cm)	79.88 ± 7.54	82.38 ± 11.38	65–92	65–106	0.407^a^
Fasting Blood Glucose	87.97 ± 24.87	90.66 ± 57.62	59.46–175	59.4–330	0.252^b^
Triglyceride	102.54 ± 49.85	155.7 ± 58.22	54.8–284.8	91–295.5	0.000^b^
Total C	167.87 ± 24.44	196.7 ± 36.72	115.2–223	113–248	0.005^a^
HDL-C	51.13 ± 7.14	37.95 ± 7.55	32.75–66.06	25–53	0.000^a^
LDL-C	96.24 ± 20.77	127.6 ± 33.45	50.96–134.2	57.7–182.5	0.001^a^

a Unpaired T-test.

b Mann-Whitney Test.

**Fig. 1:**
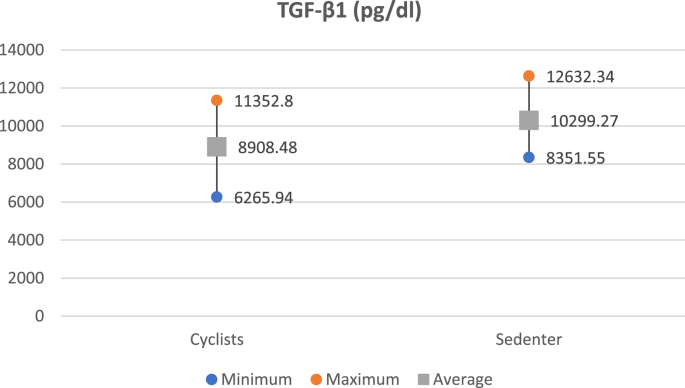
Comparison of average TGF-β1 levels between cyclists and the sedentary group.

The mean of TGF-β1 was lower in the cyclist group compared to the sedentary group (8908.48 vs 10,229.28 pg/ml). The unpaired T-test results showed that the difference was statistically significant (p = 0.001) (Fig. 1).

## 4 Discussion

The aim of this study was to compare the level of TGF-β1 between a group of recreational cyclists and a group of individuals with a sedentary lifestyle. The recreational cyclists group was a member of the bicycle communities in Mataram City who regularly rode bicycles two-to-three times a week for 30–60 min, or covered the distance of 20–40 km each time.

The nutritional status of the study subjects was represented by the body mass index (BMI) and body fat percentage. There was no significant difference between the two groups in terms of BMI and body fat percentage. Both groups had normal BMI according to the WHO standard (18.5–24.9 kg/cm2) and had average body fat percentage (18–24%) according to the American Council on Exercise. Hence, the nutritional statuses of the two groups were optimal. Several studies found correlation between BMI and TGF-β1 concentration. Scaglioneet al*.* Studied 58 hypertensive patients in ambulatory care found that BMI had a moderate, positive correlation with TGF-β1 (r = 0.52, p = 0.0001) [9]. A cohort study by Lin et al*.* in 9142 subjects in Japan had a similar finding. They conclude that serum TGF-β1 was significantly correlated with BMI (p < 0.0001). Subjects with BMI ≥25 kg/m^2^ showed higher TGF- β1 compared to those whose BMI <18,5 kg/m^27^.

The results of measuring waist circumference in the two groups gave similar results, although the average waist circumference of the control group was slightly higher than that of the cyclist group. Several studies showed a relationship between the level of TGF-β and obesity. Farhangi et al. in 84 female subjects aged 20 to 50 concluded that body mass index and waist circumference were potent positive predictors of serum TGF-β concentration (p = 0.023 and p = 0.001) and also serum TGF-β was positively associated with percent fat mass (r = 0.25, p = 0.03)and negatively with fat free mass (r = - 0.24, p = 0.04) [10]. This was based on the involvement of TGF-β in the development of adipose tissue. In addition, several studies demonstrated association between obesity phenotype and TGF-β. Alessiet al. found high expressions of TGF-β mRNA in obese individuals, and a strong correlation between the level of TGF-β antigen of adipose tissue and BMI [11]. In a case control study by Alameey et al. obese or overweight group of children was compared with healthy, non-obese children. The finding showed that the level of TGF-β1 were significantly positively correlated with the body weight, BMI (kg/m^2^) and BMI z-score of obese/overweight cases, respectively (P < 0.001, in all) [12]. Other studies in human subjects showed that secretion of TGF-β1 by adipose tissue increases in obese individuals [13].

The lipid profile (TG, Total C, HDL-C and LDL-C) significantly differs between the cyclist and the sedentary group in this study (Table 1). Studies have shown that physical exercises help to improve the lipid profile. Physical exercises lead to ±6% reduction of TG, ± 3% reduction of Total C, ± 4.5% reduction of LDL-C and ±1% increase in HDL-C [[14], [15], [16]]. In a study of 111 sedentary, overweight men and women with light to moderate dyslipidemia, Krauss et al. found that regular physical exercises and minimal reduction in body weight led to improvement of the lipid profile [15].

The levels of TGF-β1 in the cyclist group were lower than the control group and this difference was statistically significant (p = 0.001). TGF-β1 is one of the 3 beta TGF isoforms in mammals. TGF-β1 is able to elicit a variety of cellular responses, including extracellular matrix synthesis and metabolism [17]. Serum levels of TGF-β1 are associated with physical activity and exercise. Research conducted by Czarkowska-Paczek et al., concluded that serum TGF-β1 levels increased 2.73 times from 20.58 ng/ml to 55.37 ng/ml immediately after strenuous exercise and 1.95-fold, to 40.03 ng/ml, 2 h post strenuous physical exercise [18]. This increase is a response to physical exercise. Similar research conducted by Heinemeier in plasma samples, showed an increase in TGF-β1 levels from 992 pg/ml to 1301 pg/ml after the study subjects ran for 1 h on a treadmill at a 3% inclination [19].

When a person does physical exercise or sports, there will be an increase in the need for energy, which is obtained through both aerobic and anaerobic metabolism. Subsequently, leading to an increase in the formation of ROS. ROS is the result of normal metabolism utilizing oxygen [20]. At low concentrations, physiologically ROS functions as “redox messengers” in intracellular signaling and regulation, whereas at high concentrations it will induce oxidative modification of cellular macromolecules, inhibit protein function, and can cause cell death [21]. Overproduction of ROS can cause oxidative damage, however, at the same time stimulates antioxidant defense and repairs against oxidative damage [22].

TGF-β1 can increase the production of mitochondrial ROS in various cell types, by inducing NADPH oxidase and suppresses antioxidant enzyme activity, leading to a redox imbalance. On the other hand, a redox imbalance due to increased ROS and/or decreased antioxidants will activate TGF-β1 in the latent form and induce TGF-β1 gene expression, which causes increased TGF-β1 activity [6].

Physical exercise or exercise that is done regularly will induce adaptation. This adaptation process is mainly due to the increase in antioxidant activity and the enzymes that play a role, especially superoxide dismutase (SOD), catalase (CAT), and glutathione peroxidase (GPX) [20,23]. This increase in antioxidant enzymes will be able to counteract the effects of free radicals, ROS or in other words, the production of ROS can be minimized. Low levels of ROS will suppress the activation of the latent form of TGF-β1 and will lead to the low levels of TGF-β1 in serum/plasma as well. Exercise adaptations lead to metabolic changes [23], changes in neuromuscular recruitment patterns during exercise, and tissue remodeling [24]. The specific types of changes that occur after training depend on the type of stimulus, which includes: model, intensity, and volume of exercise [23].

Based on this study, regular physical activities, either by cycling or with other sports can improve physical fitness. Consequently, good physical fitness leads to the improvement of health status. However, in this study, the intensity and distance travelled by cycling were not considered.

The limitation of this study is the research sample is still limited to single city in Indonesia and the total sample is not large number (42 sample) and the sample collection time is relatively short, which isa cross-sectional design. The advantages of this research are this research is the first in Lombok Island, Indonesia and the results of this study can be used as a standard reference for Exercise Adaptation in Cyclists, especially for Indonesian and Asian people. Need research with a larger sample size in the future and multicenter. The writing of this script follows the rules of the PROCESS 2020. The work has been reported in line with the PROCESS 2020 [25].

## 5 Conclusion

The levels of TGF-β1 in the recreational cyclist group were lower than the sedentary group. Regular physical exercise will trigger an adaptation process that will strengthen the work of the antioxidant enzymes so that they suppress the activation of latent TGF-β1. In addition, regular physical exercises are also associated with better lipid profiles.

## Ethical approval

All procedure for research has been approved by Ethics Commission Faculty of Medicine, Mataram University.

## Sources of funding

No funding or sponsorship.

## Author contribution

IAEW, AA, II, and IP wrote the manuscript and participated in the study design. IAEW, HK, GWB, DPS, and RHA drafted and revised the manuscript. IAEW, GWB, and DPS performed examinations each sample of research. IAEW, AA, II, IP, HK and RHA performed bioinformatics analyses and revised the manuscript. All authors read and approved the final manuscript.

## Trial registry number

1. Name of the registry: http://www.researchregistry.com. Registration Date: August 21, 2021 15:09
2. Unique Identifying number or registration ID: researchregistry7083
3. Hyperlink to your specific registration (must be publicly accessible and will be checked): https://www.researchregistry.com/browse-the-registry#home/registrationdetails/61210919cbffd2001f7aa516/

## Guarantor

Rohadi Muhammad Rosyidi.

## Consent

This manuscript data from primer data of subjects research in Mataram University Hospital, consisted of 2 groups, each consisting of 21 participants, namely the recreational cyclist and the sedentary group. Anthropometric examinations were carried out, including body weight, height, body mass index, waist circumference, and body fat percentage.

## Disclosures

The authors report no conflict of interest.

## Provenance and peer review

Not commissioned, externally peer-reviewed.

## Declaration of competing interest

The authors declare that they have no conflict of interests.
